# Group B Streptococci and *Trichomonas* vaginalis infections in pregnant women and those with spontaneous abortion at Sanandaj, Iran

**Published:** 2018-06

**Authors:** Amjad Ahmadi, Fariba Farhadifar, Masoome Rezaii, Farnaz Zandvakili, Fariba Seyedoshohadaei, Mozhdeh Zarei, Sholeh Shahgheibi, Rashid Ramazanzadeh, Daem Roshani

**Affiliations:** 1 Social Determinants of Health Research Center, Research Institute for Health Development, Kurdistan University of Medical Sciences, Sanandaj, Iran; 2 Department of Microbiology, Faculty of Medicine, Kurdistan University of Medical Sciences, Sanandaj, Iran; 3 Department of Obstetrics & Gynecology, Faculty of Medicine, Kurdistan University of Medical Sciences, Sanandaj, Iran; 4 Master of Midwifery. Mph of Reproductive Health, Vice Chancellor for of Research and Technology, Kurdistan University of Medical Sciences, Sanandaj, Iran; 5 Cellular and Molecular Research Center, Research Institute for Health Development, Kurdistan University of Medical Sciences, Sanandaj, Iran

**Keywords:** Group B Streptococci, Spontaneous abortion, *Trichomonas vaginalis*, PCR

## Abstract

**Background and Objectives:**

Group B Streptococcali (GBS) is an important factor in newborn deaths in developed and developing countries. *Trichomoniasis* is one of the most prevalent sexually transmitted diseases (STDs) in the world, which is caused by protozoan *Trichomonas vaginalis (T. vaginalis)*. The present study compares the frequency of GBS and *T. vaginalis* genital infections in pregnant women, women with spontaneous abortion, as well as its role in spontaneous abortion.

**Materials and Methods:**

In this case-control study, 109 women were included with spontaneous abortion with gestational ages between 11–20 weeks and 109 pregnant women with gestational ages between 35–37 weeks in Sanandaj, Iran. DNA was extracted by endocervical swabs and subjected to PCR assays. The independent t-test was used; and for comparing other qualitative variables in each group, the Chi-Square Test was used.

**Results:**

The age of the women ranged from 19–43 years (29.6 ± 5.9) and in the control group the age range was from 19–42 years (27.8 ± 4.87). The rate of prevalence of Group B Streptococcal infection in the control group was 3.6%; and in the patient group there were 7.2% with the rate of prevalence of *T. vaginalis* in both groups as zero.

**Conclusion:**

The present study showed that there is no relationship between GBS infections (P-value = 0.235) and *T. vaginalis.*

## INTRODUCTION

GBS is an important factor in the death of newborns in developed and developing countries. About 10–40% of pregnant women with bacteria colonized in the body are carriers of bacteria in the rectum and vagina; and 70–80% of these women transfer the bacteria to a newborn (1, 2). The disease caused by this infection in newborns is divided into two categories: pre-term and post-term labor. In the early type, it is presumed that the disease is caused by uterine infection or when the newborn is passing through the vagina, which will be revealed after 24 h from the time of birth and sometimes up to one week later (3). Three clinical manifestations of this infection in infants are: pneumonia, meningitis, and septicemia (4). Conducted studies have also shown that the rate of antibiotic resistance to this bacterium is increasing (5, 6). *Trichomoniasis* is one of the most prevalent STDs in the world, which is caused by the protozoan *T. vaginalis*. Becoming infected by *T. vaginalis* in women may cause the inflammation of vagina, cervix, and the urinary tract (7). It is estimated that about 10–50% of infections caused by *T. vaginalis* in women are asymptomatic (8). This disease, in addition to creating symptoms in the genital tract, causes premature delivery, low birth weight (LBW), and increased mortality in infants (9). Due to the lack of information on the prevalence of these infections in pregnant women and those who have experienced spontaneous abortion in the region this study was conducted to compare the frequency of GBS and *T. vaginalis* genital infections in pregnant women, women with spontaneous abortion, and its role in spontaneous abortions in Sanandaj, North West of Iran.

## MATERIALS AND METHODS

This case-control study was performed on 109 women with spontaneous abortion with gestational ages of 11–20 and known as the “case” group; and 109 pregnant women with gestational ages of 35–37 weeks and known as the “control” group that had shown no symptoms of abortion.

Having sexual activity and not taking any antibiotics were inclusion criteria. Immunocompromised persons, people with chronic diseases (diabetes, endocrine disorders, high-blood pressure), history of repeated abortions, and traumatic and anatomic abortions were exclusion criteria. In addition to asking about the date of the first day of their most recent menstruation, ultrasound scan tests were done to estimate the gestational age of the fetus.

Research tools in this study where a questionnaire and performing PCR on cervical swab samples. First, all women signed an informed-consent form to participate in this study and after completing the questionnaire, two cervical swabs were taken from each person (one sample for freezing and the other for extraction). Then, samples were immediately placed inside 15 ml tubes that contained 5 ml of phosphate-buffered saline (PBS) buffer and were kept under −70°C until used for DNA extraction. DNA was extracted using High Pure PCR Template Preparation Kit (Roche, Mannheim, Germany). After DNA Extraction using the above-mentioned kit, the DNA samples were kept in 1.5 ml microtubes at −20°C until the PCR was performed.

Primers for tageting *T. Vaginalis* were Tvk 3: attgtcgaacattggtcttacctc and Tv*k*7: tctgtgccgtcttcaagtatgc) (10) and for GBS, were (Sag59: TTTCACCAGCTGTATTAGAAGTA) and (Sag190: GTTCCCTGAACATTATCTTTGAT) (11).

The PCR amplification program for *T. vaginalis* was: initial denaturation at 94°C for 5 min, followed by 35 cycles of denaturation at 94°C for 1 min, annealing at 61°C for 45 secs, extension at 72°C for 2 min; and final extension at 72°C for 5 min. PCR products were separated by electrophoresis in 1% gel agarose, stained by ethidium bromide, visualized by ultraviolet (UV) light and then photographed. The PCR positive control was DNA extracted from

**Fig. 1.**
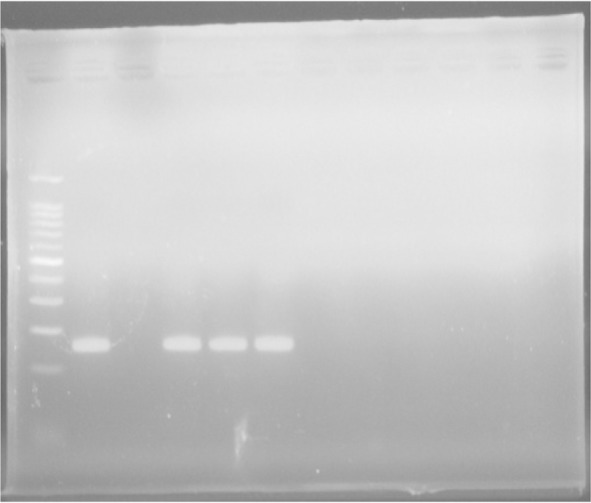
PCR for detection of Group B streptococci: Lane 1) 100 bp DNA Ladder (Cinna Clon), lane 2) PCR positive control (153 bp), Lane 3) negative control, Lanes 4–6) positive PCR products

**Fig. 2.**
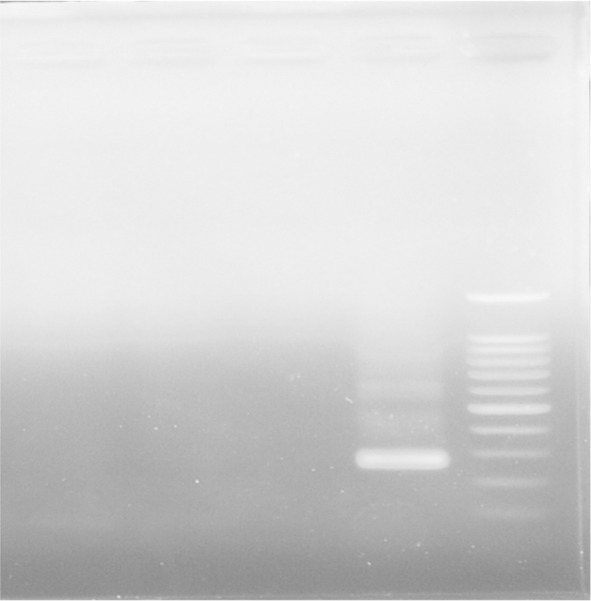
PCR test for *Trichomonas Vaginalis* detection: Lane 1) 100 bp DNA Ladder (Cinna Clon), lane 2) PCR positive control (261 bp), Lane 3) negative control, Lanes 4 and 5) negative PCR products

*T. vaginalis*. In addition, for a definite diagnosis the amplicon was sequenced. The PCR amplification program for GBS was: Initial denaturation at 94°C for 3 min, followed by 35cycles of denaturation at 94°C for 1 min, annealing at 55°C for 1 min, extension at 72°C for 1 min; and final extension at 72°C for 5 min. PCR products were separated by electrophoresis in 2% gel agarose, stained by ethidium bromide, visualized by UV light and then photographed (Figs. 1 and 2). The PCR positive control was the DNA extracted from GBS. In addition, for a definite diagnosis, its sequence was determined. For comparing the mean age in each group and to find out if they are normal, the independent t-test was used, and for comparing other qualitative variables in each group, the Chi-Square Test was used. For all the steps, significance was considered at a level of 5%.

## RESULTS

In this study, the rate of prevalence of GBS infection in the control group was four persons (3.6%), in the patient group was eight persons (7.3%), and the rate of prevalence of *T. vaginalis* in both groups was zero. In the case group, the age of the women ranged from 19–43 years (29.6 ± 5.9); and in the control group, the age range was from 19–42 years (27.8 ± 4.87). The rate of smoking in the control group was zero and in the patient group it was three (2%). The history of vaginal infection in the control group was five (4.5%) persons and in the patient group it was 11 (10%) persons. The consumption rate of alcoholic drinks in both groups was zero. The prevalence of urinary tract infection (UTI) in the control group was eight (7.3%) persons and in the case group it was nine (8.3%) persons (Table 1).

**Table 1.** Demographic data, risk factors in women with spontaneous abortion (case group) and women with normal delivery (control group)

**Variables**		**Spontaneous abortion** **n = 109**	**Normal delivery** **n = 109**	**p-value** 0.83
**Age**		(29.6 ± 5.9)	(27.8 ± 4.87)	

**Education**	Illiterate	5 (4.5%)	3 (2.7%)	
	Primary education	49 (45%)	33 (30%)	0.08
	High school education	36 (33%)	44 (40%)	
	Academic education	19 (17.4%)	29 (26.6%)	

**Occupation**	Housekeeper	97 (89%)	97 (89%)	0.99
	Employee	12 (11%)	12 (11%)	

**History of smoking**		0 (0%)	3 (2.7%)	0.43
**History of Preterm delivery**		4 (3.6%)	0 (0%)	0.044
**History of preterm premature rupture of the membranes**		5 (4.5%)	1 (1%)	0.084
**History of Vaginal infection**		11 (10.1%)	5 (4.5%)	0.115
**History of Urinary infection**		9 (8.3%)	8 (7.3%)	0.801
**Prevalence of GBS**		8 (7.3%)	4 (3.6%)	0.23

## DISCUSSION

The present study showed that the prevalence of GBS infection in the control group was four (3.6%) persons (7.2%), in the patient group was eight persons, and the prevalence of *T. vaginalis* in both groups was zero. In a study that utilized the molecular method on 400 pregnant women in Papua New Guinea, the prevalence of infection with *T. vaginalis* was reported as 21.30% (12). In another study from Mexico, the prevalence of *T. vaginalis* among 252 pregnant women was 23.40%. (13). In another study on 268 pregnant women from USA, the prevalence of *T. vaginalis* in sawb samples were reported as 16% using the molecular method (14). The prevalence of GBS in Iran, Tehran city, was reported as 9.30% and 11.20% (n= 375) among pregnant women using culture and PCR respectively (15). Another study in China, GBS in 74 women who had experienced abortions and 62 women who never had an abortion was detected. This test was performed using cervical swab samples and culture method. According to results, the rate of prevalence in the infected persons was 12.16%; and in healthy people it was 9.60%. But they were not able to find a significant relationship between GBS and abortions, P = 0.662. In this study, like our study, showed that there was no significant relationship between abortions with GBS infection (16). In a further study in USA, GBS in 212 colonized women was detected using the molecular method. GBS observed in 126 (59.4%) of them (17).

The present study showed that there is no relationship between GBS infections (P-value = 0.235) and *T. vaginalis*; and the low prevalence of infection may be indicating good hygiene services in the women of this region, which is due to visiting obstetricians and gynecologists adequately before and during pregnancy. STDs are some of the most prevalent infectious diseases in societies and inflict a heavy financial burden on the patients and society. The World Health Organization (WHO) has estimated that there are 330 million new cases of STD’s annually and most of them occur in developed countries (18). In addition, STDs are one of the biggest health problems throughout the world, especially in developing countries (3). STDs with or without clinical symptoms, increase the risk of becoming infected with HIV. For this reason, the continuation of infection by STDs is a major concern for general health in many countries (19) as this protozoan can facilitate the state of infection for cancer-causing viruses or produce cancer-causing metabolites (20, 21). In addition, GBS neonatal infection is one of the most prevalent neonatal infections that can cause newborns to die even with vaccination. The global distribution of its serotypes may change over time in different geographical locations. Therefore, creating a vaccine that can be used worldwide is unlikely (5, 6, 22, 23). Therefore, it is recommended that a screening test for the detection of STDs before pregnancy with suitable diagnostic tests is better placed during the work-routine of obstetricians and gynecologists to decrease the rates of these types of infections in society.

## CONCLUSION

The present study showed that there is no relationship between GBS infections (P-value = 0.235) and *T. vaginalis*; and the low prevalence of the above-mentioned infections is indicating a good health system service in the women of this region, which is due to visiting obstetricians and gynecologists adequately before and during pregnancy.
